# Investigating the Perceived Link Between Intercultural Mentoring and Cultural Competence Among Graduate Students and Faculty

**DOI:** 10.1002/yd.20669

**Published:** 2025-06-04

**Authors:** Bolanle Adebayo, Hannah M. Sunderman

**Affiliations:** ^1^ Department of Agricultural, Leadership, and Community Education Virginia Tech Blacksburg Virginia USA

## Abstract

Intercultural mentoring relationships, which are increasing in higher education, require cultural competence to be effective and successful. Therefore, the current study focused on perceptions of cultural competence and intercultural mentoring effectiveness among graduate students and faculty in departments of Agricultural Leadership, Communication, Education, and Extension (ALCEE) and leadership educators in the United States. Using an online survey, 32 participants shared their perceptions of mentoring effectiveness and cultural competence. The findings from the thematic analysis revealed a perception of intercultural mentoring as aiding the development of cultural competence by providing an experiential learning platform where participants learned intercultural relationship skills (e.g., how to deal with cultural differences). In addition, lessons learned through intercultural mentoring were perceived as transferable to other intercultural relationships. Our findings inform specific recommendations for intercultural mentoring training.

## 1 Introduction

The demands of the global workforce necessitate that leadership educators incorporate skills for navigating intercultural relationships when engaged in intercultural mentoring relationships and leadership development curricula (Eden et al. 2024; Nopas and Kerdsomboon 2024; Turner et al. 2019). Previous scholarship has revealed cultural competence as an important skill for navigating intercultural relationships (Adebayo and Sunderman 2025; Perry and Redman 2023; Punti and Dingel 2021; thus, prioritizing the development of students’ cultural competence is essential to success. Leadership educators, due to their direct access to leadership training opportunities for students and leaders, have the potential to be at the forefront of students’ cultural competence development through effective mentoring (Genao 2016; Rahimi and Oh 2024).

Opportunities for leadership educators to mentor students are becoming increasingly intercultural due to the globalization of education, the recent rise in e‐learning platforms, and expanding options for virtual mentoring (Goodwin 2020). Nonetheless, the existence of mentorship connections does not ensure their efficacy. Due to the cultural differences between mentors and mentees, intercultural mentoring relationships frequently encounter difficulties beyond the typical challenges of mentoring, including emotional strain. When these are not managed appropriately, they yield more harm than good (Jokikokko and Uitto 2017; Kochan and Pascarelli 2012; Madrid et al. 2016). Therefore, it is crucial for leadership educators to develop techniques and competencies (e.g., cultural competence) for successful intercultural mentoring.

Notably, leadership educators’ development of cultural competence is necessary for intercultural mentoring relationships to succeed (Desai et al. 2018; Osula and Irvin 2009; Rhodes 2002). More so, culturally competent leadership educators serve as models for students' cultural competence development, since a successful intercultural mentoring relationship can potentially improve students’ behavioral cultural intelligence and diverse relational abilities of mentors and mentees (Adebayo and Sunderman 2025; Hagqvist et al. 2020; Young et al. 2018). Additionally, the cultural competence development of leadership educators enables their understanding of the diverse needs of diverse students, thus equipping them with the knowledge and understanding valuable for developing culturally responsive leadership education curricula (Barakat et al. 2021).

In sum, intercultural mentoring tactics affect the degree to which cultural competence can be acquired. Conversely, the efficacy of intercultural mentoring may be determined by the degree of cultural competence. Responding to the call for a quantitative study on the relationship between cultural competence and mentoring effectiveness in higher education and empirical observation of the bi‐directional relationship between cultural competence and intercultural mentoring (Adebayo and Sunderman 2025), we investigated the relationship between the perceived mentoring efficacy of intercultural mentoring relationships and cultural competence.

## 2 Methods

A multimethod approach was used to examine the relationship between cultural competence and perceived intercultural mentoring effectiveness among leadership faculty and graduate students. This type of research design utilizes two or more data sources or methods to examine a research question (Lewis‐Beck et al. 2003). Data were collected through an online survey of quantitative measures and open‐ended questions. The data were analyzed to understand the perceived relationship between cultural competence and intercultural mentoring effectiveness.

### 2.1 Data Collection and Measures

Our population of interest was leadership educators, faculty, and graduate students in the Departments of Agricultural Leadership, Communication, Education, and Extension (ALCEE) in the United States. The inclusion criteria for the study were that participants must have been involved in any intercultural mentoring relationship for at least a year before data collection. The inclusion criteria and the informed consent form were written in bold on the first page of the online survey. Participants were reached through the Association of Leadership Educators (ALE) listservs, North American Colleges and Teachers of Agriculture (NACTA) listservs, and faculty and graduate students within the authors’ networks.

Cultural competence was measured with Domenech Rodríguez et al.’s (2022) cultural competence scale, while mentoring effectiveness was measured using the Berk et al. (2005) scale. Open‐ended response questions were based on the intercultural mentoring and cultural competence literature. Specifically, Adebayo and Sunderman's (2025) model elucidated a bidirectional relationship between cultural competence and intercultural mentoring relationships and guided the inclusion of questions such as, “What biases or prejudices did you have about your mentor or mentee's culture that have changed or been reinforced because of your intercultural mentoring relationship?” and, “What did you learn in a particular intercultural mentoring relationship that helped you in other intercultural experiences?”

### 2.2 Data Analysis

The quantitative data for the study were analyzed using descriptive and inferential statistics. Descriptive statistics analyzed participants’ demographic characteristics, while inferential statistics, specifically Spearman‐Rho correlations, were used to examine the relationship between cultural competence and mentoring effectiveness. The use of Spearman‐Rho correlations was considered appropriate for the study because the cultural competence and mentoring effectiveness scales utilized Likert‐type questions, which are ordinal scales (Berk et al. 2005; Domenech Rodríguez et al. 2022; Puth et al. 2015).

Thematic analysis was utilized to analyze open‐ended questions through a six‐stage process (Nowell et al. 2017). The first stage involved identifying emerging meanings and patterns. The second stage generated codes representing emerging meanings, and the third stage categorized codes into overarching themes. In the fourth stage, the categorization of codes and the themes created in the third stage were reviewed. In the fifth stage, the themes were refined to capture the emerging meanings from the findings, while the sixth stage involved reporting the findings.

## 3 Results

We used a multi‐method approach to data collection via a survey, which included quantitative and qualitative results. The quantitative and qualitative findings are discussed independently before being integrated.

### 3.1 Quantitative Results

Descriptive statistics indicated that participants were primarily leadership educators who worked in ALCEE departments (see Table 1). Further, descriptive statistics revealed race/ethnicity as the most common area of cultural difference identified by participants as present in their intercultural mentoring relationships, followed by gender. Participants were split between faculty and graduate students. Inferential statistics revealed no significant relationship between perceived cultural competence and mentoring effectiveness (see Table 2).

**TABLE 1:** Descriptive characteristics of participants.

Variables	Frequency (%)
Position in mentoring relationship	
Faculty	15 (46.9%)
Graduate student	17 (53.1%)

Work in an ALCEE department	
Yes	29 (90.6%)
No	3 (9.4%)

Area of work	
Leadership education	20 (62.5%)
Agricultural extension	3 (9.4%)
Agricultural communication	5 (15.6%)
Agricultural education	4 (12.5%)

Cultural difference(s) in intercultural mentoring relationships	
Gender difference	16 (50.0%)
Race/ethnicity difference	29 (90.6%)
Religious difference	3 (9.4%)
Sexual orientation difference	2 (6.3%)

**TABLE 2:** Relationships between cultural competence and mentoring effectiveness.

Analysis by participant group	*r* value	*p* value
Analysis among all participants	0.102	0.606
Analysis among faculty	0.043	0.879
Analysis among graduate students	0.211	0.489

*Note*: If *p* value is > 0.05, the relationship is non‐significant.

### 3.2 Thematic Analysis of Qualitative Data

A thematic analysis of open‐ended response questions revealed significant themes, including the origin of mentoring relationships, concerns prior to intercultural mentoring relationships, intercultural mentoring leading to cultural competence, challenges encountered in intercultural mentoring relationships, cultural competence training, and lessons learned that have assisted other mentoring relationships. The emergent themes provided insight into intercultural mentoring relationships among leadership educators, graduate students, and programmatic units (e.g., departments, graduate programs). The themes encourage leadership education for faculty to understand the approach needed to maximize intercultural mentoring for cultural competence in a learning environment.

#### 3.2.1 Origin of Intercultural Mentoring Relationship

Thirty (approximately 94%) of the 32 participants discussed the origin of their intercultural mentoring relationship. Sixteen participants mentioned that the start of the identified intercultural mentoring relationship was by choice, 15 mentioned it was by appointment, and four mentioned both. One graduate student explained that having mentors by appointment and choice was limited by the available mentors in their field. He said, “Arguably, within agricultural education, an intercultural relationship is both mandatory and chosen. The field is dominated by White men and women and deeply Eurocentric.” Similarly, a faculty member described the process of being paired with an intercultural mentee: “It was by choice, the potential [student] reached out to me, and we had several conversations before she applied to the graduate program.”

Another leadership faculty member detailed some mentoring relationship appointments, saying, “My school has students from a spectrum of life and cultural experiences. Students are randomly assigned mentors within their major. Mentors do not get to choose who to mentor. We often mentor students from other majors and support each other.” In sum, intercultural mentoring relationships were based on choice and appointment.

#### 3.2.2 Intercultural Mentoring Concerns

Fifteen participants had no concerns before the intercultural mentoring relationship. Seven participants mentioned communication‐related concerns such as misunderstandings, clarity, and transparency in communicating goals and expectations. For example, regarding potential misunderstandings in communication, a faculty mentor said they “wondered about the potential miscommunication,” while a graduate student talked about the “potential for miscommunication and power dynamics that are impacted by cultural norms.”

Regarding clarity in communication, a graduate student mentioned accent‐related concerns and stated, “Being new to the country, I was worried about my mentor's understanding of things like my accent and non‐verbal communication.” Similarly, faculty participants shared communication challenges in intercultural mentoring relationships. One leadership faculty noted,

“In several of the cultures in which I worked as a mentor, sometimes I would make a request, and the mentee would say ‘yes’, but the real answer was ‘no’. I had to learn how to know when ‘yes’ really meant ‘no’”. In sum, differences in language, views, and possible goals were perceived as potentially threatening effective communication in intercultural mentoring relationships.

#### 3.2.3 Challenges Encountered in Intercultural Mentoring Relationships

Seventeen participants shared that they encountered challenges in their intercultural mentoring relationships, with multiple participants reiterating references and communication as significant challenges. However, participants overcame the various challenges through learning, effective communication, and openness. A leadership graduate student, “…. conducted meetings and discussed issues with the mentors regarding effective communication as a solution to challenges.” Narrating the importance of learning, a member of the leadership faculty said: “There are always challenges, but as the mentor, the person with tenure, and the person in a position of authority, I am sometimes blind to those challenges. I must be vigilant and stay in the mindset of a learner. I think that is one way to overcome the challenges—and listen.”

Another faculty member also shared the importance of open‐mindedness, saying, “Being more open and understanding that people may act or behave based on cultural backgrounds and then learning about their behavioral tendencies relative to their culture.” However, nine participants shared that challenges did not exist in their intercultural mentoring relationships, attributing this to respect for cultural differences, open‐mindedness, and willingness to learn. One faculty member noted, “No [challenges] because we see each other as whole persons and not as a specific race, gender, or pedigree. Having a high degree of respect for each other's backgrounds, values, family of origin, and cultural context helped a lot.” Taken together, learning, open‐mindedness, and effective communication helped participants to prevent or overcome the challenges encountered in intercultural mentoring relationships.

#### 3.2.4 Intercultural Mentoring as a Learning Opportunity

Twenty participants shared how cultural competence was developed during intercultural mentoring, providing opportunities for learning new things, relational skills, cultural differences, and respect. Specifically narrating how intercultural mentoring helped to develop their cultural competence, a faculty member shared:

I have learned a ton just by being close to and spending time with my GTAs. I make mistakes and try to have a relationship where my mentee can tell me what I did. We also discuss almost everything—content, how the class went, and how we are doing.

Also, sharing how cultural competence aided their intercultural mentoring, another faculty said, “I was so appreciative of my advisees' willingness to share personal stories about their upbringing and experiences—seeing things from their perspective was so instructive for my cultural competence.”

Participants shared various lessons and information learned, such as cultural differences, culturally related stories, and reflective communication, which accumulated to aid the development of cultural competence. For example, a faculty member learned to “think before I talk.” Another faculty member narrated learning via intercultural mentoring: “It showed me the need to move beyond respect into inclusion and belonging. Respect is first, but it is not enough.” Participants in intercultural mentoring relationships also played active roles in the learning experience, such as observing, being open‐minded, asking questions, and listening. For example, a leadership graduate student shared that they “…observe how my advisor relates to advisees from other cultural backgrounds (also different from my own).” In sum, participants shared experiences of how intercultural mentoring aided the development of cultural competence, albeit not in a vacuum but in cooperation with other attributes like willingness to learn and share.

#### 3.2.5 Cultural Competence Training

When asked about prior cultural competence training, participants had a variety of responses, including not having training (*n* = 7), having non‐formal training (*n* = 9), and engaging in formal training (*n* = 6) or university coursework (*n* = 4). Regarding experience with non‐formal training, a faculty member mentioned “…experience working at universities teaching students of varying cultural backgrounds and offering flexibility around culture,” as cultural competence training. Narrating their experiences as non‐formal cultural competence training, a graduate said, “[I] spent many years in and leading international student groups before this intercultural mentoring relationship with my advisor. I also had amazing bosses and supervisors from different cultural backgrounds who taught me to lead across differences and flourish.”

Another faculty member shared a similar experience. They said, “I have conducted research in cultural adaptation. I have taught school in [another country] and have had extensive international experiences in agriculture and Extension education. My experience helps the mentoring relationship because I have seen how people from different cultures view things differently. I have also learned to respect the differences between different people.”

Participants who mentioned training or coursework said the training included inclusion, reflexivity, and intercultural communication. For example, a graduate student said, “The DEI course offered in my program and many LinkedIn learning videos on related issues are helpful. The course is an interesting exposition of different diversity, equity, and inclusion concerns and how to engage in difficult conversations when necessary.” Similarly, a faculty member explicitly mentioned diverse formal and informal training forms and lessons learned. The faculty shared:

I have attended various PD seminars on cultural competence, taken the Intercultural Development Inventory, and had a certified facilitator process the results with me. Luckily for me, my closest colleague is a recognized expert in cultural competence, so I have been very blessed to have lots of informal cultural competence training via that relationship. The most important takeaways from both formal and informal cultural competence training that benefited the relationship were: (1) Always seek first to understand; (2) When something seems off‐putting, check what kind of implicit assumptions might be informing that perception; (3) Cultural competence is not about being perfectly culturally competent all the time. Cultural competence is often about having a humble and reflective spirit ‐ always learning, trying to do better, and being willing to apologize when needed.

Overarchingly, cultural competence training occurred informally through participants’ experiences and formally via training and classes.

#### 3.2.6 Lessons Learned That Have Assisted Other Mentoring Relationships

Participants mentioned skills they learned from intercultural mentoring that can be transferred to other intercultural mentoring relationships, including relational skills (*n* = 9) and managing differences (*n* = 9). Regarding relational skills, a graduate student said, “Seeing how my advisor connects to advisees from vastly different cultures has taught me how to better relate to people from different cultures.” Specifically, participants mentioned relational skills such as empathy, humility, patience, mutual respect, asking questions, and transparency.

For example, a faculty member mentioned that they “…learned to be patient because everyone works at different efficiency levels.” In the same vein, a graduate student said:

I have learned much about mutual respect, patience, and transparency as necessary forms of relationship building. Having a mentorship where those are clear is extremely helpful because there are always moments when people see or understand things differently. It is often all too often that differences in cultural background go unresolved or unaddressed when those factors are absent.

A faculty member also learned the importance of asking questions in relationships:

Intercultural mentoring has taught me so much about seeking first to understand and the importance of asking questions. Some of the wealthiest developments in my cultural competence came from just asking questions about their lives and experiences. Such a joy and blessing!

Participants mentioned learning how to engage differences through intercultural mentoring relationships. In managing differences, participants learned to accept others, avoid stereotyping, and have a learning attitude. For example, regarding avoiding stereotyping, a faculty member learned “…to utilize knowledge of cultural norms and values as a guide but not assuming all people from a particular culture will follow those patterns.” In sum, intercultural mentoring equipped participants with intercultural relational skills and knowledge of how to manage differences.

### 3.3 Integration of the Quantitative and Qualitative Data

Although the quantitative and qualitative data were collected concurrently, both types of data revealed nuances in the relationship between intercultural competence and mentoring effectiveness among leadership education faculty and graduate students. First, the inferential analyses of the quantitative data were non‐significant, indicating the lack of a statistically significant relationship between perceived cultural competence and mentoring effectiveness in intercultural mentoring relationships. However, the qualitative results revealed a process of learning cultural competence in intercultural mentoring relationships by overcoming concerns and challenges, embracing the opportunity to learn, engaging in cultural competence training, and translating the learning into other relationships and contexts. The qualitative findings emphasize how a learning attitude is necessary to navigate intercultural mentoring relationships and develop cultural competence.

## 4 Discussion

Regarding the quantitative findings, the small sample size may have limited the power of the statistical analyses. Considering the current qualitative findings, future researchers are encouraged to continue examining the relationships between mentoring effectiveness and cultural competence within intercultural mentoring relationships. Beyond the sample size, the non‐significant relationship between intercultural mentoring effectiveness and cultural competence might also be because culture is multifaceted (Barrett 2018); thus, cultural competence is multifaceted and occurs on a continuum (Cross 2012). Consequently, having cultural competence in a specific context might not guarantee mentoring effectiveness in intercultural mentoring relationships. Therefore, leadership educators are encouraged to devote themselves to continual learning and development of cultural competence

Regarding the qualitative findings, the results contribute to the field of intercultural mentoring in that (a) intercultural mentoring provides an experiential learning platform for developing the cultural competence of mentees and mentors; (b) cultural competence is essential to ensure effective learning in intercultural mentoring relationships; and (c) training could encourage the cultural competence required to navigate intercultural mentoring relationships challenges.

The results corroborated Adebayo and Sunderman's (2025) assertion of intercultural mentoring as an experiential learning platform for the development of cultural competence. Strategies for overcoming initial concerns and challenges of intercultural relationships were learned and tried within the intercultural mentoring relationship (e.g., effective communication, open‐mindedness, and respecting perspectives). Thus, intercultural mentoring relationships served as experiential learning platforms for developing cultural competence. Although participants did not specifically discuss reflection—the central theme in experiential learning—participants expressed Kolb's (1984) assertion of learning as a combination of experience, cognition, behavior, and perception through various levels of engagement (e.g., effective communication) and attitudinal behaviors (e.g., open‐mindedness). Overarchingly, the findings suggested incorporating experiential learning principles (e.g., reflection) into intercultural mentoring training. Moreover, as with all learning situations, the results showed that participant engagement, specifically communication and curiosity, was crucial for learning cultural competence in intercultural mentoring.

The field of leadership education also emphasizes the importance of reflection in facilitating development following experiential learning activities (White et al., 2016). Leadership educators are urged to view intercultural mentoring relationships as opportunities for experiential learning, thus grounding the relationships in reflection to overcome concerns and challenges. As higher education experiences an increase in intercultural mentoring relationships in response to globalization, leadership educators may utilize their expertise in learning and development to maximize the success of intercultural mentorship relationships and facilitate cultural competence among mentors and mentees.

The findings also illustrated how an initial level of cultural competence is helpful to ensure effective learning in intercultural mentoring relationships. Participants shared that previous cultural competence training and intercultural mentoring experiences helped them navigate cultural differences and associated challenges. For example, a participant shared that the cultural competence training through mandatory coursework helped them engage in difficult conversations in mentoring relationships. Such benefit illustrates that training contributes to navigating mentoring challenges since communication was one of the significant challenges of intercultural mentoring.

The results also corroborate studies recommending cultural competence training for navigating intercultural mentoring challenges (Oikarainen et al., 2022; Rahimi et al., 2023). The role of the initial level of cultural competence in learning cultural competence through intercultural mentoring is revealed in the cyclical process depicted by Adebayo and Sunderman (2025), indicating that screening for cultural competence in mentors may be helpful. In line with existing literature, the results emphasized the importance of interpersonal and attitudinal skills, such as open‐mindedness and self‐awareness, as necessary attributes to ensure active learning engagement for developing cultural competence (Miller 2007; Rhodes 2002; Sanchez and Colon 2005; Vaccaro and Camba‐Kelsay 2018). Thus, it is recommended that mentors be screened and trained to possess interpersonal and attitudinal skills before the appointment of intercultural mentees (Adebayo and Sunderman 2025). Training may be particularly impactful for leadership education faculty and graduate students to improve their effectiveness as role models and facilitators of inclusive leadership. Additionally, culturally competent leadership educators would be better equipped to design inclusive curricula that meet and address the needs of diverse leaders.

Specifically, as illustrated by participants’ experiences (or lack of experiences) with intercultural mentoring training, training on cultural competence prior to intercultural mentoring relationships is essential and underutilized in promoting successful intercultural mentoring relationships and developing cultural competence for graduate students and faculty. The findings have four specific recommendations for intercultural mentoring training, particularly in graduate leadership education programs.

1. We recommend mentors and mentees' training include context‐specific cultural competence recommendations (e.g., international graduate student and faculty advising relationships). As nearly half of the participants mentioned that intercultural mentoring relationships were due to appointments, it is possible that mentors and mentees might not be competent in the mentor/mentee's culture, thus affirming the benefits of training and discussion on specific cultural differences.
2. In addition to highlighting context‐specific cultural competence recommendations, the results encourage training on navigating challenges associated with intercultural mentoring relationships (e.g., communication). Notably, leadership education graduate programs may wish to share the thematic results of the current research to prompt discussion among intercultural mentors and mentees regarding challenges, concerns, and learning opportunities. Although the current findings emerged from leadership education faculty and graduate students in ALCEE departments, other disciplines may also find them relevant for intercultural mentoring training.
3. It is also recommended that training for intercultural mentoring relationships be grounded in the principles of experiential learning, a particularly important suggestion in the field of leadership education because of the value placed on reflection. Participants’ lessons learned through intercultural mentoring relationships highlight intercultural mentoring as an experiential learning platform (Adebayo and Sunderman 2025). Since reflection is a critical process in experiential learning (Kolb 1984), a series of reflection exercises should be facilitated for faculty and graduate students in intercultural mentoring relationships. For example, leadership educators may encourage journaling to raise awareness and internalize lessons learned in intercultural mentoring among faculty and graduate students. Mentors and mentees are encouraged to discuss their responses to the reflection questions to enhance the meaning‐making processes.
4. The results revealed communication as important for navigating intercultural mentoring relationships, including overcoming challenges associated with such relationships and maximizing the relationship for learning. Thus, it is recommended that leadership education graduate programs create structures (e.g., anonymous feedback exercises, expectations surveys, and reflection exercises) to facilitate open communication between intercultural mentors and mentees (Adebayo and Sunderman 2023). Although not explicitly formulated for intercultural mentoring relationships, leadership education faculty and graduate students may find Golde's (2010) Student‐Advisor Expectation Scales helpful in elucidating productive conversations on expectations.

## 5 Limitations and Future Research Directions

First, the current study, particularly the quantitative component, was limited by a smaller‐than‐optimal sample. Although the sample size was sufficient to run the analyses, additional participants would add power and allow for considering related variables (e.g., years spent in the intercultural mentoring relationship, type of interculturality [e.g., Gender, country of origin]). Future studies on the perceived relationship between cultural competence and intercultural mentoring among leadership education faculty and graduate students with a larger sample size are encouraged.

Second, we acknowledged how culture is multifaceted (e.g., gender, race, country of origin) and gave participants options to select their type of intercultural mentoring relationship. However, the analysis of participants’ responses did not consider the different types of intercultural mentoring relationships because of sample size limitations. Third, the scales used to collect data on mentoring effectiveness and cultural competence were not designed to be specific to a particular type of intercultural mentoring relationship. Future studies on a particular culture or intercultural mentoring situation (e.g., domestic faculty advising international graduate students) may provide more specific, in‐depth results and recommendations.

Fourth, the results reveal that cultural competence assisted in navigating intercultural mentoring challenges; thus, the current study recommends experimental studies of the effect of cultural competence training of graduate students on the success of intercultural mentoring relationships, as against when the training is focused on faculty. Finally, participants described how mentees’ willingness to share culturally related stories aided their learning. Thus, future research on the role of storytelling, particularly on the part of mentees, is encouraged (Adebayo and Sunderman 2023).

In sum, the current article revealed that prior cultural competence in intercultural mentoring assists mentees and mentors in navigating intercultural mentoring challenges, thus aiding intercultural mentoring effectiveness. Additionally, intercultural mentoring served as an experiential learning platform for developing cultural competence for mentors and mentees through the relational experience. Therefore, leadership educators, both graduate students and faculty, are encouraged to introspectively use the opportunity to learn and develop cultural competence to effectively prepare students. In so doing, leadership graduate programs may aid faculty and students in developing cultural competence and inclusive leadership.
